# Dementia-specific adaptations to physical performance tests of balance, mobility, and lower limb strength and function: a reliability study in people with dementia

**DOI:** 10.1186/s12877-025-06710-1

**Published:** 2025-11-15

**Authors:** Bettina Barisch-Fritz, Jelena Krafft, Janina Krell-Roesch, Alexander Woll

**Affiliations:** Institute of Sports and Sports Science, Karlsruhe Institute of Technology, Karlsruhe, Germany

**Keywords:** Motor performance, Motor tests, Physical performance, Psychometric properties

## Abstract

**Background:**

Valid and reliable physical performance tests are crucial for accurately assessing the physical performance of people with dementia (PwD) and for evaluating the effects of interventions. However, existing physical performance tests for PwD often show insufficient reliability. This study aims to investigate the reliability of physical performance tests of balance, mobility and lower limb strength and function that were specifically adapted for PwD.

**Methods:**

We conducted a reliability study with test-retest design and a one-week gap between tests among PwD living in nursing homes. Adaptations were made to either the instruction and administration, or the scoring of the three physical performance tests as follows: The Frailty and Injuries: Cooperative Studies of Intervention Techniques (FICSIT) for balance (adaptations to administration and scoring), the Timed-Up and Go Test (TUG) and its five phases (standing up, walking, turning around, walking, sitting down) for mobility (adaptations to instruction, administration and scoring in terms of analysing the five phases separately, and the Sit-to-Stand test (STS) for lower limb strength and function (adaptations to instruction and administration). The tests were standardized in terms of cues used to administer and guide them. We assessed absolute reliability (Standard Error of Measurement; Coefficient of Variance, Minimal Detectable Change) and relative reliability (Intraclass correlation coefficient; weighted Cohen’s Kappa). The absolute and relative test-retest reliability of the PP tests was assessed.

**Results:**

We examined relative and absolute reliability values of PP tests in a sample of 26 PwD (mean age, 88 years; mean Mini Mental State Examination (MMSE) score, 14). No statistically significant differences were found between baseline and retest. Relative reliability values ranged from 0.258 to 0.505 for balance (FICSIT), 0.011 to 0.860 for mobility (TUG), and 0.506 to 0.678 for lower limb strength and function (STS). Absolute reliability values as indicated by the coefficient of variation (CV) ranged from 23.5 to 92.8.

**Conclusions:**

Adaptations regarding test administration and/ or scoring did not improve reliability values as compared to the original test versions. TUG test phases showed the highest reliability values for the gait phases. Future adaptations should focus on reducing the cognitive component of demand during physical performance tests. Assistive technologies such as augmented reality could improve test reliability by providing more consistent and controlled test environment.

**Trial registration:**

German Clinical Trials Register DRKS00019205, retrospectively registered, registration date: 11 November 2019.

## Background

Physical activity interventions have shown promising effects in improving cognitive and physical function and slowing disease-related decline in people with dementia (PwD) [1–4], albeit the overall level of evidence is low-to-moderate [5]. There are several reasons for this lack of evidence, including a limited number of randomized controlled trials (RCTs) with low risk of bias/confounding [5]; peculiarities due to the target population such as heterogeneity with regard to participants characteristics; or limitations and differences associated with the quality and quantity of physical activity interventions [6]. Furthermore, when evaluating the effects of physical activity interventions on the physical performance of persons with cognitive impairment due to dementia, the quality criteria of physical performance tests are highly relevant for correctly assessing potential changes resulting from the intervention.

Research has also shown that the absolute reliability, i.e. measurement error variance, is unsatisfactory for assessing physical performance [7]. Although commonly used motor performance assessments such as the Timed Up & Go Test, and Functional Reach Test demonstrated sufficient relative and inter-rater reliability (ICC >0.75), their absolute test-retest reliability was often inadequate for detecting individual-level change in PwD [7].

A valid and reliable assessment of physical performance in PwD is also related to the cognitive performance of a person, and may differ between dementia subtypes [8]. Poor physical performance is associated with an increased risk of dementia and Alzheimer’s disease [9], and the use of physical parameters for diagnostic purpose has thus been discussed in the literature [10]. In addition, physical performance is highly relevant to daily care and care needs [11, 12], e.g., reduced mobility leads to increased care needs [13]. Most nursing home residents with dementia have significant physical limitations and often experience a decline in physical performance after admission to the nursing home [14]. To ensure that this decline is adequately monitored and preferably addressed by targeted intervention and therapy, physical performance should be assessed routinely on admission [14].

Physical performance tests for PwD typically focus on physical abilities such as static and dynamic balance, mobility, strength, endurance and flexibility [7]. The validation and evaluation of tests to assess physical performance in PwD is of great importance to ensure their suitability for use in PwD. The quality criteria of physical performance tests in PwD were examined in a systematic review by Trautwein and colleagues [7]. The review revealed insufficient research on validity [7]. However, inter-rater and relative test-retest reliability were generally acceptable with intraclass correlation coefficients (ICC) ranging from 0.75 to 0.95. In contrast, absolute test-retest reliabilities were frequently not acceptable, as indicated by high coefficients of variation (CV >20%) and elevated standard error of measurement (SEM) values. Absolute reliability reflects the consistency of individual scores over time and is commonly quantified using the minimum detectable change (MDC) at various confidence levels (e.g. MDC_95_) [15, 16]. In contrast, relative reliability provides information about the consistency of the measurement in heterogeneous target populations. It is usually expressed using the ICC [17]. The ICC indicates a test’s ability to differentiate between participants and reflects individuals’ positions relative to others in the group. However, it does not provide information about the accuracy of results for individuals.

Test-retest reliability is also influenced by the use of external cues to compensate for cognitive impairment [7]. Furthermore, the degree of cognitive impairment may influence the actual physical performance test results [18], thus making it difficult to validly assess physical performance in PwD. In addition, due to the frequently identified balance problems in PwD [19] and the high variance within balance performance [20], both ceiling and floor effects limit the sensitivity with regard to test quality. A pilot study involving 15 community-dwelling older adults with mild to moderate Alzheimer’s disease, found good test-retest reliability and moderate inter-rater reliability for Berg Balance Scale [21]. However, the MDC_95_ was relatively high, indicating a lack of sensitivity in the physical performance tests. The authors concluded that the clinical utility of the Berg Balance Scale for PwD might be limited by memory-related difficulties, as well as comprehension issues resulting in inconsistent performance of the tasks, which further biases the reliability. This indicates insufficient sensitivity in the tests or scales, which also leads to bias in assessing potential changes due to physical activity interventions.

In their study, Chan and Pin investigated the reliability, validity, and MDC_95_ of the 2- and 6-minute and 10-meter walking tests in 99 frail PwD [22]. Despite the relatively high MDC_95_ values, they concluded that the tests were reliable. They also examined the provided cues and identified a general consistency in the cueing system. However, they also noted that participants’ performance was influenced by motivation and fatigue, and concluded that such inconsistent factors in PwD could lead to variability in test outcomes that may not accurately reflect their physical ability.

Overall, this suggests that physical performance tests need to be adapted for PwD. This has been partially addressed by cueing systems designed to make the tests easier for participants to understand and complete [22]. However, cueing, which is generally effective, highlights a central problem: standardized protocols may not be applicable to all dementia patients. The need for individualized support can lead to variability and thus alleviate generalizability.

There are several technologies, such as wearable devices, that may offer opportunities to monitor physical performance and collect more comprehensive data to assess physical performance [23], and which may also allow a reduction in cognitive demands during the physical performance tests. Further studies are needed to evaluate their potential for standardized assessment procedures in this population [24].

The type of adaptation required may depend on the test itself, the form of support and standardization currently provided, and the scoring system. The necessary form of adaptation to the conditions of PwD and its potential contribution to evidence-based decision-making has not yet been sufficiently discussed in the literature. Therefore, this study was conducted to gain an initial insight into the possibilities and necessity of this approach. To the best of our knowledge, only few studies to date have focused on adaptations of physical performance tests to dementia-specific conditions, i.e., cognitive impairments, and no study has examined the reliability of these adaptions, which can be identified as a relevant research gap. The aim of this study was therefore to investigate the reliability of physical performance tests adapted for PwD. The adaptations were made to either (a) the instructions and administration, or (b) the scoring procedures of the physical performance tests. We hypothesised that the relative reliability of the selected tests would be good (ICC > 0.75), and that the absolute reliability would change due to the adaptations. The results of this study will provide important information on the reliability of physical performance and their adaptations in a sample of PwD.

## Methods

### Study design and procedure

We conducted a reliability study to assess the relative and absolute test-retest reliability of standard and adapted physical performance tests for balance, mobility, lower limb strength and function in PwD. The study adheres to the Declaration of Helsinki and was approved by the Ethics Committee (blinded). It was registered it in the German Clinical Trials Register (blinded). Data collection took place in February and March 2020.

We assessed the reliability of physical performance tests focusing on static and dynamic balance, mobility, and lower limb strength and function. All participants completed the physical performance tests once at baseline (R1) and once at retest (R2), with a seven-day interval between test sessions and without prior practice. There were no additional interventions during the interim period. The test conditions were standardized with regard to the examiners (i.e., trained sports scientists), the test location, the time of the day, and the order of the tests. The physical performance tests were administered in two blocks – one block before and one block after lunch. Participants were randomly assigned to one of the two blocks to avoid bias, e.g. due to fatigue. Also, examiners and participants were blinded to the previous test results, i.e., they did not have access to baseline results during the retest.

### Sample and recruitment

Participants were recruited from four nursing homes in Southwestern Germany. All participants or their legal guardians received full information about the study and gave informed consent prior to the study. Inclusion criteria were a diagnosis of mild to moderate dementia due to Alzheimer’s disease, vascular dementia, or other primary dementia (as confirmed by a physician), Mini Mental State Examination (MMSE) score < 24, age 65 years or older, ability to walk at least 10 m with or without a walking aid, and general practitioner’s clearance. Exclusion criteria included other severe neurological disease, other severe acute disease, and/or severe physical impairment.

### Primary and secondary outcomes

The physical performance tests used in this study were the Frailty and Injuries: Cooperative Studies of Intervention Techniques (FICSIT) [25] for balance, the Timed-Up and Go Test (TUG) [26] for mobility, and the Sit-to-Stand test (STS) [27] for lower limb strength and function. All tests were standardized in terms of the cues used to administer and guide the tests. For all original versions of the tests, instructions were used as stated in the original sources. For the purpose of this study, adjustments were then made to the instructions and administration, for example, by simplifying and segmenting the instructions to reduce cognitive load and improve task comprehension, and by standardizing the test administration through consistent verbal cues or alignment to everyday tasks to aid orientation and minimize variability in performance. The scoring procedures were also refined for functional subtasks (e.g., in the FICSIT and TUG) to reduce interpretive ambiguity and better capture individual performance. All tests were shown once and one test run was allowed.

#### Frailty and injuries: cooperative studies of intervention techniques (FICSIT)

Static balance was assessed using the FICSIT [25]. In this test, participants are asked to perform four different standing positions for 10 s: (1) Romberg, (2) semi-tandem, (3) tandem, and (4) single-leg stance. The time for each position is measured manually. The original scoring system requires each position to be held for 10 s (FICSIT_original_). The scoring system is based on scores between 0 and 5, with 5 being the maximum score that can be achieved, meaning that all tasks can be performed and held for 10 s. In order to account for the large variance in the sample, FICSIT was also performed with an additional task (FICSIT-A_original_), namely lateral abduction of the dominant arm to shoulder height with a dumbbell (male: 1.0 kg, female: 0.5 kg) in all described positions.

For both performances (FICSIT and FICSIT-A), the original scoring system was used, as well as an adapted version based on the distinction of how long each position can be maintained (less than 3 s vs. 10 s) (see Table 1).

**Table 1:** Original and adapted scoring of FICSIT

	Original scoring for FICSIT_original_ and FICSIT-A_original_ according to (Rossiter-Fornoff et al. 1995)	Adapted scoring for FICSIT_adapted scoring_ and FICSIT-A_adapted scoring_
0	Subject refused, failed, or was excluded from parallel stance	Not feasible, normal standing position for < 3 s
0.5	Parallel position for < 10 s	Normal standing position for < 10 s but > 3 s
1	-	Normal standing position for10 sec
1.5	Parallel standing position for 10 s, or semi-tandem position for < 10 s	Parallel standing position for < 10 s but > 3 s
2	Semi-tandem position for 10 s, refused, failed, or excluded from tandem position	Parallel standing position for 10 s
2.5	-	Semi-tandem position for < 10 s but > 3 s
3	Semi-tandem position maintained for 10 s, or tandem position for < 10 s	Semi-tandem position for 10 s
3.5	-	Tandem position for < 10 s but > 3 s
4	tandem position for 10 s or one-legged position for < 10 s	Tandem position for 10 s
4.5	-	Single-legged position for < 10 s but > 3 s
5	Single leg position for 10 s	Single-legged position for 10 s

Two adaptations were made. Firstly, the administration was modified by adding a dumbbell that had to be held with the arm extended. This focused participants’ attention on the held object, but also made the task more challenging. Secondly, the scoring system was modified because the FICSIT shows significant variation in balance performance among PwD. This suggests that more sensitive intermediate steps need to be defined and assessed.

An additional outcome measure has been added for both FICSIT and FICSIT-A. This outcome is the total time in which each position can be maintained.

#### Timed up & go test

Mobility was assessed using the TUG [26]. The TUG was administered by asking participants to stand up from a chair, walk 3 m around a cone, walk back 3 m, and sit back down on the chair. The time taken to complete the task was recorded as in the original version. The chair had armrests and a seat height of 46 cm. The use of walking aids was allowed. The adapted version (TUG-A) was administered in the context of a familiar everyday activity involving sitting in a chair and walking to a specific destination. For TUG-A, participants were asked to retrieve a standard remote control located in front of a television set, just behind the three meters marked by a cone. They were then asked to walk back and sit on the chair again. The familiar, everyday nature of the administered adapted version helps to address issues with understanding and logical thinking, and reduces nervousness and uncertainty during the testing situation.

In both versions (TUG and TUG-A) additional time measurements were defined based on Porciuncula et al. [28]. The task was divided into 5 phases TUG_P1−P5_, TUG-A_P1−P5_:

P1: Getting up from the chair - time from the start of getting up from the chair to crossing the start line.

P2: Walking three meters - time from crossing the start line to the cone line.

P3: Turning around the cone - time from first to second crossing of the cone line before and after the cone.

P4: Walking three meters back - time from the cone line to the start line.

P5: Sitting down on the chair - time from crossing the start line to sitting on the chair.

Subdividing TUG into phases provides more insights into both mobility as well as cognitive impairments which may be masked when only measuring the total TUG time. Verbal cuing for the test instruction was further standardized to: “Stand up” (TUG)/“Get the remote control” (TUG-A); “Walk around the cone” (only TUG); and “Sit down on the chair” (TUG and TUG-A). The verbal cue was given once, at the beginning of the test. If the participants stopped before the 3 m or did not comply to the verbal cues, a second trial was initiated. Time was measured using a handheld stop watch.

#### Sit-to-Stand test (STS)

The modified STS [27] was used to assess lower limb strength and function. Participants were asked to perform the STS task as many times as possible in the given time of 30 s and 60 s. In addition, the time taken to stand up from the chair five times was recorded. The chair had armrests and a seat height of 46 cm. The modified version of the STS allowed the participants to use the armrests to stand up [29]. Adaptions to the STS administration were made by giving participants acoustic signal when they reached the standing position with maximum hip extension (STS-A_30sec_, STS-A_60sec_, STS-A_5x_). The acoustic signal (beep sound), which was activated using infrared light barriers (height level of the participant’s forehead, hip angle approximately 180°), was given in full standing position to indicate that the repetition is valid and the participant can sit down again. This adjustment was perceived as helpful as it gave participants additional acoustic feedback, reminding them of each completed task and improving their retention. Additionally, instructors counted the repetitions out loud.

#### Secondary outcomes

Secondary outcomes included general cognition using the MMSE [30], body mass index (BMI; weight using Seca 813 Robusta scale, height using Seca 213 stadiometer (Seca, Hamburg, Germany)), sex and age. In addition, we asked physicians to provide information on health data (diagnosis of dementia, etiology of dementia, number of medications, and Cumulative Illness Rating Scale (CIRS) [31]). We also documented the use of walking aids during all physical performance tests.

#### Statistical analysis

Results were tested for normal distribution, and test and retest values were compared using paired t-test or Wilcoxon test to rule out systematic bias. All statistical calculations were performed using IBM SPSS version 29 (IBM Corporation, Armonk, USA). A two-tailed p-value less than 0.05 indicated statistical significance.

Relative reliability was assessed using the ICC and weighted Cohen’s Kappa. For the ICC calculation, we used two-way mixed effects model based on single measurement type and absolute agreement with a 95% confidence interval [32]. For ordinal scores, we calculated weighted Cohen’s Kappa and reported both linear and quadratic weights to better describe the shape of the disagreement distribution [33]. According to the COSMIN criteria for good measurement properties we considered ICC or weighted Kappa ≥ 0.70 as sufficient for group comparisons [34]. Individual comparisons were assessed according to Koo and Li [32]: ICC < 0.5 as poor, ICC = 0.5–0.75 as moderate, ICC = 0.75–0.90 as good, and ICC >0.90 as excellent.

Absolute reliability was assessed by the SEM, the CV, and the MDC_95_ [17, 35, 36]. The interpretation of the SEM and MDC_95_ is based on the original unit of the test values. The MDC_95_ can be considered as an estimate of the responsiveness of a measurement [37] to assess and interpret effects and thus identify ‘true’ changes.

In general, a smaller SEM corresponds to better absolute reliability [15]. The CV can be used to compare absolute reliability between tests as it is a dimensionless value [17]. The interpretation of the CV depends on the purpose of the test and the user, and is left to the practitioner, but it is recommended that acceptable values for the CV, and therefore absolute reliability, should be 10% or less [38].

## Results

### Sample characteristics

26 participants with a mean age of 88 (SD 5) years and a mean MMSE of 14 (SD 5, range 5–24) were included in the reliability study. Table 2 presents the characteristics of the participants.

**Table 2:** Sample characteristics of participants at baseline

Characteristics	Total sample, [*n* = 26]
	M (SD), range
MMSE, *N* = 25	14 (5), 5–24
Age, years, *N* = 26	88 (5), 77–97
CIRS, *N* = 19	
Morbidity Index	8 (7), 2–33
Severity Index	1.3 (0.3), 1–2
	not available 27% [*n* = 7]
Number of medications, *N* = 20	8 (5), 1–17
BMI, kg/m², *N* = 23	29 (3), 20–36
	N (%)
Sex, female N (%)	19 (73)
Type of dementia, N (%)	
Alzheimer’s disease	9 (35)
Vascular dementia	4 (15)
Mixed dementia	5 (19)
other	1 (4)
unknown	7 (27)
Use of walking aid, N (%)	
walker	16 (62)
no walking aid	10 (39)

*BMI* Body Mass Index, *CIRS* Cumulative Illness Rating Scale, *df* degree of freedom, *M* mean, *MMSE* Mini-Mental State Examination, *n* number, *SD* Standard deviation

The absolute and relative test-retest reliability of the physical performance tests are shown in Table 3. Paired t-tests and Wilcoxon tests showed no statistically significant differences between baseline (R1) and retest (R2) for all physical performance tests, either in the original or adapted forms (data not shown).

**Table 3:** Absolute and relative test-retest reliability of adapted physical performance tests

	*N*	R1		R2		Difference |R2-R1|	Cohen’s weighted Kappa^l/q^ /ICC (95% CI)	SEM	CV	MDC_95_
		Median (IQR)* Mean (SD)	Range	Median (IQR)* Mean (SD)	Range					
FICSIT_adapted scoring_ [points]	22	2.5 (2.1)*	0–4.5	3.0 (1.6)*	0–4.5	1.0	0.258 (−0.020-0.536)^l^ 0.450 (0.109–0.791.109.791)^q^	1.0 0.9	39.1 33.7	2.8 2.4
FICSIT_l_ [points]	22	1.5 (2.8)*	0–4	2.0 (1.8)*	0–4	1.0	0.327 (0.054–0.599.054.599)^l^ 0.500 (0.185–0.814.185.814)^q^	1.1 0.9	52.9 45.6	2.9 2.5
FICSIT [time in s]	22	26.3 (13.1)	0–46	27.3 (10.5)	0–46	9.0	0.496 (0.097–0.756.097.756)	8.3	31.7	23.1
FICSIT-A_adapted scoring_ [points]	17	2.5 (1.8)*	0–4	2.5 (1.5)*	0–5	0.8	0.414 (0.097–0.730.097.730)^l^ 0.505 (0.043–0.968.043.968)^q^	1.0 0.9	41.7 38.3	2.6 2.4
FICSIT-A [points]	18	1.5 (1.9)*	0–4	1.5 (1.9)*	0–5	0.9	0.366 (0.047–0.684.047.684)^l^ 0.441 (0.033–0.848.033.848)^q^	1.0 1.0	57.2 53.7	2.9 2.7
FICSIT-A [time in s]	17	23.4 (13.0)	0–40	23.9 (12.6)	0–50	8.3	0.523 (0.056–0.799.056.799)	8.7	37.1	24.1
TUG [s]	15	28.5 (15.5)	11.9–65.8	26.1 (15.2)	11.0–59.9	8.6	0.673 (0.268–0.876.268.876)	8.6	30.3	23.9
TUG_P1_ [s]	15	3.1 (2.7)	1.1. −10.6	2.9 (2.2)	1.0–9.1	1.9	0.460 (−0.070-0.782)	1.8	57.7	5.0
TUG_P2_ [s]	15	6.1 (4.2)	1.9–18.7	6.0 (3.5)	2.7–14.2	1.6	0.860 (0.631–0.951.631.951)	1.4	23.5	4.0
TUG_P3_ [s]	15	6.8 (5.7)	1.4–20.3	5.2 (4.6)	1.8–16.0	5.0	− 0.036 (−0.539-0.475)	5.2	76.3	14.5
TUG_P4_ [s]	15	6.8 (3.1)	3.0 −13.5	7.3 (4.7)	3.5–20.7	2.9	0.338 (−0.215-0.719)	3.2	47.5	8.9
TUG_P5_ [s]	15	5.6 (4.2)	0.7–16.7	4.8 (3.4)	1.2–13.3	2.4	0.700 (0.326–0.887.326.887)	2.1	36.5	5.7
TUG-A [s]	16	29.5 (15.6)	12.0–70.7	26.0 (15.9)	12.9–77.8	10.6	0.365 (−0.146-0.721)	12.4	42.1	34.4
TUG-A_P1_ [s]	15	3.9 (3.6)	1.1–15.4	3.1 (2.9)	1.2–12.9	2.5	0.282 (−0.260-0.685)	2.7	70.9	7.6
TUG-A_P2_ [s]	15	6.3 (3.6)	3.4–15.6	5.8 (2.5)	3.4–11.1	1.8	0.431 (−0.099-0.766)	2.3	36.8	6.4
TUG-A_P3_ [s]	15	8.4 (7.7)	1.5–21.6	6.6 (8.2)	1.3–35.2	6.2	0.011 (−0.521-0.516)	7.8	92.8	21.6
TUG-A_P4_ [s]	15	6.9 (4.6)	2.9–19.2	6.8 (3.2)	3.5–13.7	2.7	0.445 (−0.095-0.775)	2.9	42.1	8.1
TUG-A_P5_ [s]	15	4.4 (3.6)	0.7–15.3	4.1 (2.6)	1.0–10.0	1.9	0.508 (−0.006-0.805)	2.2	49.4	6.0
STS-A_5x_ [s]	12	23.4 (13.7)	9.8–59.0	24.0 (14.5)	10.5–51.0	9.2	0.678 (0.181–0.896.181.896)	7.8	33.5	21.7
STS-A_30s_ [n]	12	7.8 (3.6)	3.0–14.0	7.8 (3.3)	3.0–13.0	2.6	0.506 (−0.102-0.831)	2.4	30.6	6.6
STS-A_60s_ [n]	12	15.1 (7.1)	6.0–29.0	15.0 (5.9)	6.0–24.0	4.6	0.600 (0.041–0.868.041.868)	4.0	26.7	11.2

*CI* Confidence interval, *CV* Coefficient of variance, *FICSIT* Frailty and Injuries, Cooperative Studies of Intervention Techniques, *ICC* Intraclass Correlation Coefficient, *m5x STS* modified five times Sit-to-Stand Test, *m30sSTS* Modified 30 s Sit-to-Stand Test, *m60 STS* Modified 60 s Sit-to-Stand Test, *N* number, *PwD* People with dementia, ^q^ quadratic,* R*1 baseline assessment, *R*2 retest, *SD* Standard deviation, *SEM *Standard error of measurement, *CV* Coefficient of variance, *TUG* Timed Up & Go Test

The range of relative reliability values is between 0.258 and 0.505 for FICSIT, between 0.011 and 0.860 for TUG, and between 0.506 and 0.678 for STS. The absolute reliability values of the calculated CV, which allow comparison between tests, ranged from 23.5 to 92.8. Values for relative reliability showed highest values for the phases 2 and 5 of the TUG.

## Discussion

The study did not reveal statistically significant differences in physical performance tests, i.e., balance, mobility, lower limb strength and function, between baseline and pretest. In addition, no relevant differences were found between the types of adaptation, i.e., instruction and administration or scoring.

The relative reliability values are poor (ICC < 0.05) for all physical performance tests. Even the point estimate which was in the range of moderate for the TUG and for the time of phase 5 in both the original and adapted TUG are poor as their lower bound of the 95% confidence interval for the ICC is below 0.5 [32] Relative reliability values are only good (ICC = 0.860) for phase 2 of the original TUG. The absolute reliability values are above 10% for all tests and variants of physical performance and must therefore be considered unacceptable [38].

Prior research has reported better reliability values for balance, mobility and lower limb strength and function. For example, a systematic review [7] concluded that the relative test-retest reliability of balance, as assessed by the FICSIT in one study, was adequate, while absolute test-retest reliability was indeterminate or unacceptable. Relative test-retest reliability for mobility, as assessed by the TUG in 16 studies included in the review, showed adequate overall reliability, but with a wide range from no evidence to strong evidence, and absolute test-retest reliability was also low or very low [7]. For lower limb strength and function, relative test-retest reliability, as assessed by 30 s STS in 5 studies and 5xSTS in 7 studies, was adequate to moderate, but absolute test-retest reliability was again indeterminate or unacceptable [7]. A single study found high values for relative test-retest reliability in persons with Alzheimer’s disease, with two test sessions separated by only 30–60 min of rest [39]. Most studies used test-retest between-day design in PwD [7].

The reliability values in this study are rather low, compared to a study by Blankevoort et al., who reported higher reliability values for mobility (TUG; 2.12 SEM, 5.88 MDC_95_), balance (FICSIT; 0.55 SEM, 1.52 MDC_95_), lower limb strength and function (30sSTS; 1.26 SEM, 3.49 MDC_95_) assessed in 58 cognitively impaired people [18]. One potential explanation for the reduced reliability values may be the composition of our sample, which included participants who were more severely affected by dementia compared to participants in Blankevoort et al. [18]. Indeed, an impact of the level of cognitive function was also reported by Blankevoort et al. with lower reliability in more affected participants [18]. The authors further concluded that the physical performance tests evaluated are useful for detecting differences, but are less suitable for monitoring clinically relevant intra-individual performance changes [18]. This limitation was even more pronounced in our sample, where the absolute reliability values (e.g., SEM, MDC) were lower than previously reported. The extremely high CV values observed in certain subcomponents (e.g., TUG-AP3 = 92.8%) may reflect underlying floor or ceiling effects, thus limiting the sensitivity of these tests to detect change. Moreover, day-to-day variability in PwD, such as fluctuations in mood, fatigue, or attention, may have further contributed to inconsistent test performance and elevated measurement error. These findings underscore the need for adapted assessments and stratified analyses to better account for variability in dementia severity. Overall, our study contributes to the literature by highlighting the challenges of assessing absolute reliability of physical performance tests in persons with more severe cognitive impairment.

In general, it has been shown that examining individual phases of the TUG adds value to the assessment. This has also been reported in other studies [40]. In particular, the two walking phases have a high degree of reliability. However, although these walking phases provide information about overall mobility, they do not provide information about transfer performance, which is increasingly important in old age and in individuals with increasing care needs. The fact that the walking phases are reliable is consistent with the results of gait analysis, which also tends to reveal good values for the reliability of walking and various gait parameters in PwD [7].

Interestingly, our attempts to improve the reliability of physical performance tests using the two adaptation strategies, i.e., changes to (a) instruction and administration or (b) scoring of the physical performance tests, did not lead to an improvement in reliability scores. While it is unknown as to why the adaptations did not result in better reliability scores, we may speculate that the adaptations did not sufficiently decrease the complexity of the task or the instructions; and adapting the physical performance test to an everyday situation (e.g., retrieve a remote control placed in front of a television set instead of the original TUG) also showed no improvement.

It has been postulated that various factors may have a significant influence on physical performance and the reliability of physical performance tests in PwD, including but not limited to the degree or severity of cognitive impairment [18]. This raises the question as to whether the cognitive demands of physical performance tests may be masking motor demands or may be too challenging for the task to be successfully completed by PwD. In many physical performance tests, performance in different cognitive domains such as attention and processing speed, but also memory and executive functions, may contribute to the test results [41]. It can therefore be assumed that a reduction of these cognitive demands, which might be achieved by the use of additional, technical tools such as augmented reality, e.g., to provide visual aids such as arrows on the floor, could have a beneficial effect on reliability scores. In addition, factors that depend on daily fluctuations related to e.g., time of the day, type and stage of dementia, or environmental factors, and which are common in the PwD may also play an important role for physical performance test reliability [42, 43]. In addition, differences in physical performance performance between two measurement points may also be related to an actual decline in physical performance performance in PwD. In some cases, performance may decline relatively quickly over the course of several days or few weeks which is characteristic for the, at times, rapid progression of dementia [44, 45]. By considering the possible influence of cognitive demands and daily fluctuations, amongst other factors, other studies may have found better reliability values for within-session, followed by within-day, and between-day designs than our study.

### Strengths and limitations

One strength of the study is the high standardization of the physical performance test administration, and we deliberately put high emphasis on an administration identical to the original version with the same frequency and wording of the cues in the adapted tests. However, this does not seem to have had a sufficient effect on the reliability scores, and thus on reducing a possible cognitive impact.

One major limitation that needs to be considered when interpreting the results is the relatively small sample size, which did not allow for subgroup analyses to explore the potential impact of cognitive impairment on the reliability of physical performance tests. The limited number of participants likely contributed to wide confidence intervals and may have led to an underestimation of reliability values, particularly for ICCs, which are sensitive to sample variance [46].

This restricts the generalizability of our findings and limits the strength of conclusions regarding the reliability of the test adaptations. Furthermore, drop-outs occurred across all tests, with some participants missing one of the two assessments or declining participation. This is an issue which is commonly reported in studies involving PwD [47], potentially influenced by daily fluctuations in mood or motivation. These factors collectively reduce the interpretability and robustness of our results.

## Conclusions

Measuring physical performance in PwD is a major challenge. In general, geriatric physical performance tests that have not been developed specifically for PwD are often used [7]. Weaknesses in these tests are particularly apparent during administration and are reflected in moderate to low reliability values. In this study, an attempt was made to modify and improve physical performance test reliability values by making adjustments to (a) the instruction and administration, or (b) the scoring. However, we observed that reliability values were rather poor and could not be improved by the adjustments. This suggests that the tests and particularly the adjustments made did not sufficiently account for cognitive impairment in PwD. We postulate that the cognitive demand during physical performance tests may interfere with test execution and may mask actual physical performance. The observed high absolute measurement error, reflected in elevated SEM and CV values, has important clinical and methodological implications. It limits the tests’ ability to detect meaningful intra-individual changes, which limits usability for monitoring interventions effects and individual progression. In order to truly measure physical performance in PwD, adjustments would need to be made to effectively reduce the possible impact of cognitive demands during the tests. While this study focused on test-retest reliability, content and construct validity were not formally assessed. The adaptations were based on clinical expertise and prior feasibility work; however, further validation is needed to confirm whether the adapted tests appropriately measure the intended constructs in PwD. Certain test components, such as TUG phase 2, showed comparatively better reliability and may offer promise for future adaptations.

Furthermore, new technologies such as augmented reality may be utilized to increase the prompting nature of a physical performance test and to guide and control test execution with various overlays rather than verbal instructions alone. Another solution could be to refrain from the classical test situation and assess physical performance more directly in everyday life. This could be achieved through technical solutions such as different sensors or assistive technologies such as exoskeletons that could be used not only for support purpose but also for recording and analyzing physical performance parameters in real life.

## Data Availability

All data and materials pertaining to this study is available upon request via the corresponding author in the form of cumulative tables. Due to the sensitivity of the data, access to the raw data is not possible.

## Abbreviations

AD: Alzheimer’s disease
ADL: Activities of daily living
CIRS: Cumulative Illness Rating Scale
CV: Coefficient of variation
FICSIT: Frailty and Injuries: Cooperative Studies of Intervention Techniques–4
ICC: Intra-class correlation coefficient
MDC_95_: Minimal Detectable Change within 95% confidence interval
MMSE: Mini Mental State Examination
PwD: Persons with dementia
STS: Sit-to-Stand Test
TUG: Timed-Up and Go Test
